# Prothèse totale inversée bilatéral de l’épaule: à propos de deux

**DOI:** 10.11604/pamj.2015.20.272.6107

**Published:** 2015-03-20

**Authors:** Hassan Boussakri, Ihab Alassaf, Samir Hammoudi, Mohammed Elidrissi, Mohammed Shimi, Abdelhalim Elibrahimi, Abdelmajid Elmrini, Jean François Dumez

**Affiliations:** 1 Service de Chirurgie de Traumatologie et d'Orthopédie (AP4), Hôpital De Moulins-Yzeure, Moulins, France; 2 Service de Chirurgie Osteoarticulaire (B4), CHU Hassan II, Université Sidi Mohammed Ben Abdellah, Fès, Maroc

**Keywords:** Omarthrose, ostéonécrose, épaule, sœurs gémellaires, prothèse inversée, glenohumeral osteoarthritis, osteonecrosis, shoulder, twin sisters, reverse prosthesis

## Abstract

Omarthrose bilatérale de l’épaule sur ostéonécrose est une pathologie rarement traitée dans la littérature. La présence de cette pathologie chez deux sœurs gémellaires n'est jamais décrite. Sa prise en charge chirurgicale représente un défi et nécessite un chirurgien orthopédique expérimenté en chirurgie du membre supérieur. Nous présentent deux sœurs gémellaires qui avaient une omarthrose bilatérale de l’épaule sur ostéonécrose, qui ont été traités dans notre département par prothèse inversée. La planification préopératoire a été réalisée par une équipe de chirurgiennes entrainées. Globalement nos résultats (cliniques et radiologiques) immédiats, moyen terme et au dernier recul étaient satisfaisants. Le but de notre travail est d'attirer l'attention sur cette association rare et de discuter sa prise en charge thérapeutique.

## Introduction

La prise en charge de l'ostéonécrose de la tête humérale est peu rapportée en littérature, alors quelle représente la deuxième localisation après la tête fémorale qui est par contre largement étudiée [1]. La grande différence anatomique et fonctionnelle entre les deux articulations coxo-fémorale et scapulo-humérale expliquent en grande partie le retard des manifestations cliniques de la localisation humérale, en effet les charges que subissent la scapulo-humérale sont moins important, ce qui rend l'ostéonécrose de la tête humérale asymptomatique et le diagnostic se fait généralement au stade avancé. Par ailleurs l'arthroplastie totale de l’épaule représente un choix thérapeutique surtout dans les cas avancés. Les auteurs rapportent deux observations cliniques d'une ostéonécrose bilatérale de l'articulation scapulo-humérale, chez deux patientes, sœurs gémellaires, âgés de 69 ans, sans notion de traumatisme. L'objectif de ce travail est de présenter cette association lésionnelle rare, ainsi que notre expérience à travers ces deux observations et insister sur l'intérêt de traitement chirurgical par arthroplastie. Globalement, au dernier recul, nos résultats anatomo-cliniques sont excellents.

## Patient et observation

### Observation numéro 1

**Figure 1:**
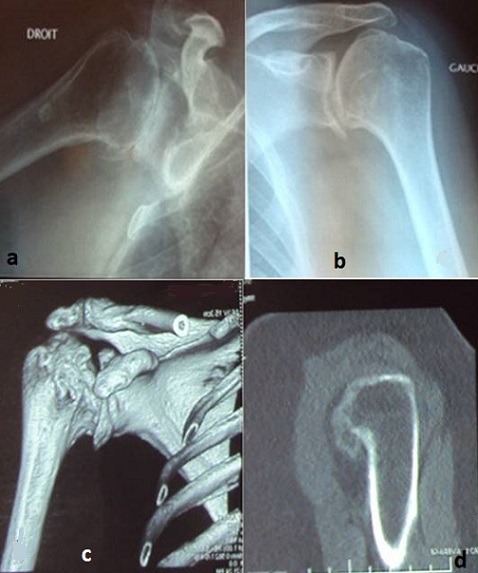
(a,b) la radiographie standard numérique des deux épaules a objectivé une omarthrose avancée de l'articulation glèno-humérale, classé IV; (c,d) la TDM des deux épaules avec reconstruction bidimensionnel (2D) et tridimensionnel (3D)

Madame CO âgée de 69 ans, secrétaire médicale de profession, retraitée, droitière, sans antécédent pathologique, notamment pas de prise de corticoïdes, qui présente depuis 5 ans des douleurs bilatérales d’épaule avec EVA chiffré à 7 droite et 6 à gauche, en dehors de tout traumatisme, ni pathologie inflammatoire connue. La symptomatologie s'est aggravée par la perte de l'abduction active des deux épaules ce qui a motivé la patiente à consulter au sein de notre formation spécialisée du membre supérieur pour prise en charge. L'examen clinique a trouvé une patiente en très bon état générale, l’épaule est d'aspect normal sans amyotrophie du deltoïde avec limitation des amplitudes articulaires des deux épaules. Le secteur de mobilité était: abduction à 70° à droite et 40° à gauche, antépulsion à 70° à droite et 80° à gauche, retropulsion à 20° à droite et 30° à gauche, rotation externe à 20° à droite et 20° à gauche. La radiographie standard numérique des deux épaules a objectivé une omarthrose avancée de l'articulation glèno-humérale, classé stade IV selon la classification Cruess (Figure 1 a,b). Un complément scannographique par une TDM de l’épaule bidimensionnelle (2D) et tridimensionnel (3D) pour une évaluation du capital osseux de la glène et une classification précise des dégâts osseux. (Figure 1c,d)

### Observation numéro 2

**Figure 2:**
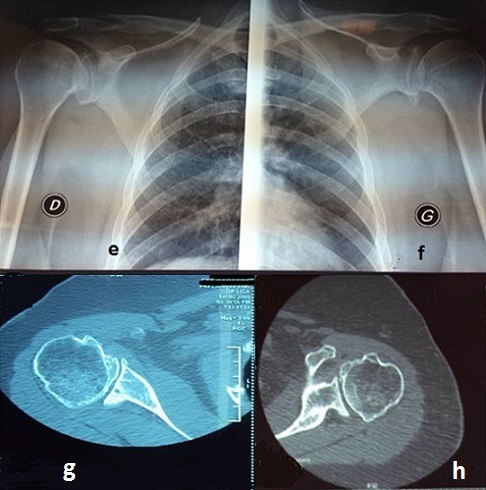
(e,f) la radiographie standard a objectivé une omarthrose avancée de l'articulation glèno-humérale classé stade V; (g,h) un complément scannographique de l’épaule bidimensionnel (2D) et tridimensionnel (3D)

Madame BM âgé de 69 ans, secrétaire médicale de profession, retraitée, droitière, sans antécédent pathologique notable. L'anamnèse étiologique n'a pas trouvé de facteur de risque, notamment pas de prise de corticoïdes, ni pathologique inflammatoire connue, qui présente depuis 4 ans, des douleurs mécaniques bilatérales des deux épaules avec une EVA chiffré à 5 à droite et 6 à gauche, en dehors de tout contexte traumatique, cette symptomatologie est devenant invalidante avec limitation de toute activité quotidienne surtout les gestes nécessitant l'abduction. L'examen clinique a trouvé une patiente en bonne état générale, autonome. A l'inspection, les deux épaules sont d'aspect normal sans amyotrophie du deltoïde, avec limitation des amplitudes articulaires des deux épaules et retentissement sur la vie quotidienne. Le secteur de mobilité était: Abduction à 50° à droite et 70° à gauche, antépulsion à 60° à droite et 70° à gauche, retropulsion à 25° à droite et 25° à gauche, rotation externe à 10° à droite et 20° à gauche. La radiographie standard a objectivé une omarthrose avancée de l'articulation glèno-humérale classé stade V selon Cruess (Figure 2 e,f). La TDM de l’épaule réalisée comme complément du bilan radiologique avec reconstruction bidimensionnelle (2D) et tridimensionnelle (3D) pour un bilan précis du capital osseux de l’épaule notamment de la glène (Figure 2 g,h). Les deux patientes ont bénéficiés d'une arthroplastie bilatérale totale de l’épaule (PTE inversée) avec un intervalle de 2 ans entre les interventions par le même opérateur au même centre hospitalier entre 2008 et 2011.

**Figure 3:**
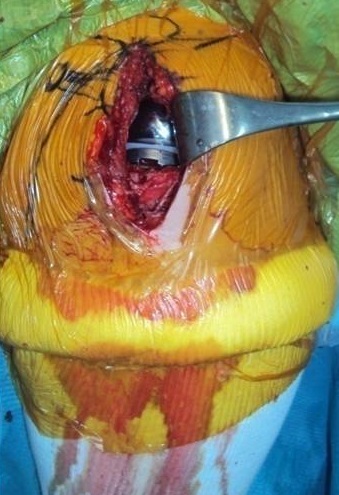
Voie d'abord externe de NEER

**Technique opératoire**: la première intervention s'est fait du côté droit chez les deux patientes. Sous anesthésie générale associé à un bloc locorégional. Sur une table ordinaire, en decubitus dorsale, position demi-assise. Le membre supérieur installé librement sur un appui-bras. Par un abord chirurgical externe type Neer (Figure 3). Dissection plan par plan, désinsertion minime du deltoïde et du bord interne de l'acromion, puis la dissection se poursuivent dans l'axe des fibres entre le faisceau moyen et faisceau antérieur deltoïdien. L’écarteur orthostatique est mis en place pour faciliter l'exposition du ligament acromio-coracoïdien réséqué. Repérage de la pointe d'entrée dans le canal médullaire 1 cm environ en dedans et 1cm en arrière de la coulisse bicipitale et la trépanation à l'aide d'une pointe carrée, alésage par tailles successifs jusqu’à l'obtention d'un bon accrochage. Positionnement du guide de coupe humérale avec une inclinaison de 135° et une rétroversion de 10°, la hauteur de la résection est établie à la jonction cartilage /os. Ensuite alésage métaphysaire réalisé par râpes de taille croissante. la rap définitive est laissée en place pour protéger l'extrémité supérieur de l'humérus. En deuxième temps, L'exposition de la glène se fait grâce à 3 écarteurs: antérieur, postérieur et inferieur et réalisation d'une capsulectomie totale. Le trou central est repéré avec le bistouri électrique qui est foré à l'aide d'une mèche suivant le guide de perçage glénoïdienne. Fraisage successif avec une petite fraise. Coupe antérieure réalisée a l'ostéotomie puis le logement de la quille centrale est préparé tout d'abord avec une mèche et suivi par des ostéotomies de l'ancillaire. Ensuite, le défenseur est ajusté exactement à la taille de la future quille par impaction de spongieux. Mise en place d'une embase de glène sans ciment taille choisi, glénosphère de diamètre adaptée. vissage de la glénosphère définitive. Ensuite cupule humérale de longueur et de taille adapté avec une tige cimentée avec du ciment au gentamycine et un insert huméral diamètre adapté avec impaction. La réduction par manœuvre externe. Dans les suites immédiates, une écharpe type bondage coude au corps est réalisée chez les deux patient enlevés à J + 2 en post-opératoire, une rééducation douce et passive a été prescrite dès le premier jour en post-opératoire active à partir de la 4ème semaine. L'examen anatomopathologique de la pièce opératoire objective une tête humérale irrégulière, l'examen microscopique de cette tête a trouvé un os non viable sous forme de zone fibreuse avec présence de cartilage périphérique sous forme de séquestre.

**Figure 4:**
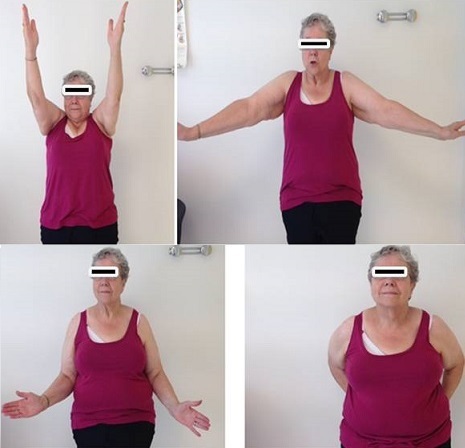
Résultats fonctionnels satisfaisants

**Figure 5:**
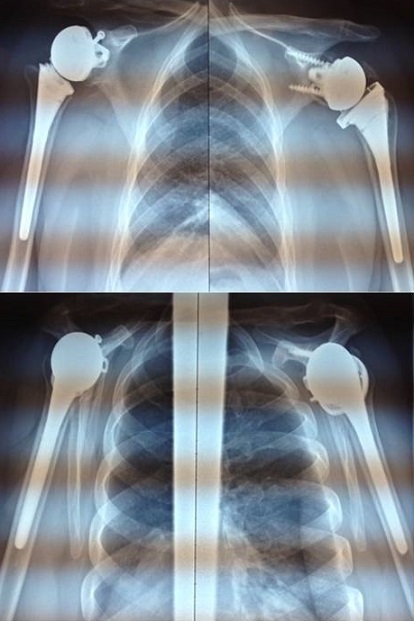
Résultats radiologiques au dernier recul

**Résultats**: nous rapportons dans ce travail le cas de deux femmes, jumelles, âgée de 64 ans, qui présentent une omarthrose avancée sur ostéonécrose de la tête humérale bilatérale isolée, associé à des lésions massives de la coiffe des rotateurs irréparables, qui souffraient des douleurs des deux épaules avec retentissement fonctionnel important, avec un score de Constant préopératoire pondéré à 40%. Les deux patientes ont été suivies en consultation régulière avec un recul moyen de 4 ans, avec un score de Constant nettement amélioré chiffré au dernier recul à 95% (Constant pondéré) (Figure 4, Figure 5).

## Discussion

L'ostéonécrose de la tête humérale représente la deuxième localisation après la tête fémorale, alors qu'elle est peu rapportée dans la littérature [1]. L'ostéonécrose humérale avasculaire aseptique peut être primaire, idiopathique, ou secondaire [1, 2]. Les facteurs de risques de l'ostéonécrose sont représentés essentiellement par la corticothérapie au long cours, l’éthylisme, la drépanocytose, la thalassémie. D'autres causes plus rares sont parfois retrouvées, telles que maladie de Gaucher, dyslipidémie, embolie gazeuse par accident de décompression. La corticothérapie au long cours, l’éthylisme, la drépanocytose peuvent être à l'origine des localisations multiples: atteinte bilatérale et/ou localisation à la hanche. Le tableau clinique de cette pathologie associe classiquement une douleur et limitation des mouvements articulaires voire enraidissement articulaire avec gène fonctionnelle et retentissement sur la vie quotidienne. La classification radio-clinique de Ficat et Arlet modifiée par Cruess [3, 4] est reconnue, par son intérêt diagnostique, évolutif et thérapeutique [5, 6]. Elle a été adoptée pour l’épaule. Le stade 1 est le stade pré-radiologique. Les symptômes se résument à une douleur non spécifique. Les radiographies simples sont normales. Le diagnostic est évoqué sur l'imagerie par résonance magnétique (IRM) devant des remaniements osseux non spécifiques. Le stade 2, correspond aux premières anomalies radiographiques visibles sur les radios simples. Peu spécifiques, elles associent plage d'ostéocondensation et d'ostéoporose. Leur localisation à la partie supérieure et postérieure de la tête humérale est évocatrice. Le stade 3, caractérise la nécrose et signe un tournant évolutif de la maladie avec l'apparition du signe de la coquille d’œuf ou “Crescent sign”, correspondant à une fracture sous-chondrale. À ce stade, la sphéricité de la tête humérale est conservée. L'ostéonécrose s'organise, prend une forme triangulaire avec une base périphérique où se situe la fracture sous-chondrale irréversible. Le stade 4, correspond à la perte de la sphéricité humérale par effondrement de l'os chondral. L'importance de l'effondrement est variable en fonction de la qualité osseuse de la tête humérale. Majeur, il peut s'agir d'un véritable collapsus osseux déstabilisant l'articulation glèno-humérale avec la médialisation significative de l'humérus. Une ostéoporose locale et un âge avancé sont des facteurs favorisant la survenue d'un collapsus osseux huméral. Le stade 5, correspond à une arthrose secondaire conséquence de la dégradation articulaire glèno-humérale.

La prise en charge des stades avancés de cette pathologie fait appel à l'arthroplastie totale de l’épaule. L'arthroplastie totale de l’épaule a bien évoluée depuis les travaux de Neer et Judet concernant les implants et les indications. Certes L'ostéonécrose avasculaire est une indication peu fréquente mais classique de la prothèse d’épaule. Le stade V de la classification de Cruess (arthrose secondaire) relève de la prothèse totale. Dans les autres stades, la prothèse humérale, voire la prothèse de resurfaçage est indiquée sauf dans le stade IV (perte de la sphéricité de la tête humérale) où les résultats ne montrent pas de différence entre prothèse humérale et totale. La rupture de la coiffe des rotateurs est un facteur péjoratif de la prothèse humérale avec survenue secondaire d'un pincement glèno-huméral. L'existence d'un collapsus osseux massif responsable d'une médialisation de l'humérus est aussi un facteur de mauvais pronostic. Devant cette forme sévère chez la personne âgée, l'indication de prothèse totale inversée s'impose. Les prothèses inversées présentent un intérêt dans cette entité pathologique surtout en cas d'association des lésions de la coiffe des rotateurs et/ou une usure et perte du capital osseux de la glène qui représente l'un des facteurs de risques de descellement des prothèses de l’épaule. En fait l'embase métallique de la prothèse inversée avec son plot central et ses quatre vis périphériques permet de se passer de ciment, et sa stabilité permet de l'implanter même lorsque le stock osseux est limité. Les contraintes qui s'exercent sur la glénosphère ne sont pas des contraintes en cisaillement, mais très rapidement au cours de l’élévation des contraintes en compression. Le système non cimenté autorise également la stabilisation d'une greffe spongieuse sous l'embase métallique, particulièrement utile en cas d’érosion centrale importante. Concernant notre travail, au dernier recul, la différence entre les deux épaules opérées n'est pas significative avec un score de Constant peu mieux sur un côté par rapport à l´autre, explique par le constat clinique préopératoire initial. Ainsi que de nombreux facteurs différents influent sur les résultats globaux en particulier l´âge, et le retard de diagnostic et de la prise en charge l´opération chirurgicale, l'expérience de chirurgien. Globalement nos résultats fonctionnels sont excellents.

## Conclusion

Omarthrose bilatérale de l’épaule sur ostéonécrose chez deux sœurs gémellaires est une pathologie orthopédique rare, jamais décrite dans la littérature. Le traitement chirurgical par arthroplastie de l’épaule à considérablement amélioré le pronostic de cette entité pathologique. Le diagnostic précoce et l´expérience du chirurgien sont indispensables pour obtenir un excellent résultat.
